# Highly enhanced ELISA sensitivity using acetylated chitosan surfaces

**DOI:** 10.1186/s12896-020-00640-z

**Published:** 2020-08-19

**Authors:** Tania García-Maceira, Fé I. García-Maceira, José A. González-Reyes, Elier Paz-Rojas

**Affiliations:** 1 Canvax Biotech; Parque Científico y Tecnológico Rabanales 21, c/Astrónoma Cecilia Payne s/n, Edificio Canvax, 14014 Córdoba, Spain; 2 Departamento de Biología Celular, Fisiología e Inmunología, Universidad de Córdoba, Campus de Excelencia Internacional Agroalimentario, ceiA3, 14014 Córdoba, Spain

**Keywords:** Chitosan surface, Chitin binding domain, Antibody orientation, ELISA

## Abstract

**Background:**

The enzyme-linked immunosorbent assay (ELISA), is the most widely used and reliable clinical routine method for the detection of important protein markers in healthcare. Improving ELISAs is crucial for detecting biomolecules relates to health disorders and facilitating diagnosis at the early diseases stages. Several methods have been developed to improve the ELISA sensitivity through immobilization of antibodies on the microtiter plates. We have developed a highly sensitive ELISA strategy based on the preparation of acetylated chitosan surfaces in order to improve the antibodies orientation.

**Results:**

Chitin surfaces were obtained by mixing small quantities of chitosan and acetic anhydride in each well of a microtiter plate. Anti-c-myc 9E10 low affinity antibody fused to ChBD was cloned and expressed in CHO cells obtaining the anti-c-myc-ChBD antibody. We found that anti c-myc-ChBD binds specifically to the chitin surfaces in comparison with anti-c-myc 9E10, which did not. Chitin surface was used to develop a sandwich ELISA to detect the chimeric human protein c-myc-GST-IL8 cloned and expressed in *Escherichia coli*. The ELISA assays developed on chitin surfaces were 6-fold more sensitive than those performed on standard surface with significant differences (p<0,0001).

**Conclusions:**

As shown here, acetylated chitosan surfaces improve the antibody orientation on the substrate and constitute a suitable method to replace the standard surfaces given the stability over time and the low cost of its preparation.

## Background

The ELISA is a powerful and widely used technique which has been used for decades to detect different molecules, especially protein analytes, in diagnostic and research context. This highly versatile technique allows the detection of biomolecules with high specificity and sensitivity, associating the readout with a subsequent enzymatic reaction producing colorimetric, fluorescence or luminescence signals [1, 2].

Disease biomarkers detection on clinical samples have great importance for diagnosis as well as for the monitoring of disorders. However, the effectiveness of the detection is frequently limited by the sensitivity and quantification capacity of the assay. Due to its high specificity and sensitivity, ELISA technique is probably the most used technique for these purposes, although for many biomarkers this technique has shortcomings based on criteria like kinetic properties and/or antibody availability [3, 4]. To cope with this issue, several methods have been developed to increase the sensitivity of the ELISA technique. In this sense, several surfaces have been designed in order to improve antibodies orientation and density [4, 5], while other methods have been established to improve the detection process by amplifying the ELISA signals [6].

In a standard sandwich ELISA assay, the analyte detection is based on the use of two specific antibodies: a primary or capture antibody, which is adsorbed to a polystyrene surface with high protein binding capacity, and a secondary antibody which generally is biotinylated. The catalyzing enzyme can be horseradish peroxidase (HRP) which normally is linked to streptavidin. The high affinity streptavidin-biotin binding facilitates the signal amplification [7].

To improve analyte detection, several methods have been developed. Among them, the use of silver nanoparticles, streptavidin-coated microparticles, or the signal amplification with the tyramide system, can be mentioned [8–10].

The adsorption of capture antibodies to standard polystyrene surfaces takes place due to hydrophobic and electrostatic interactions [11]. In this process of immobilization, the antibodies can acquire a random orientation hindering the interaction with its antigen. In addition, in the contact surface some steric impediment can occur and even the antibodies can be denatured with the consequent loosening of ability to detect the antigen. Furthermore, during the development of an ELISA the antibody can be displaced by other proteins, for example, during surface washing steps [12, 13]. All these effects can result in lower sensitivity of a large number of ELISAs with the subsequent reduced reproducibility. In order to decrease these adverse effects on antibodies immobilization, and thereby improving the sensitivity of the ELISA technique, some procedures have been developed. These methods include, for example, surface modification to make them more hydrophilic or more suitable for the covalent binding of the antibodies, and the use of high-affinity intermediate molecules [14].

The production of monolayer surfaces is one of the methods that best permit the adsorption of antibodies. In addition, the monolayers facilitate the appearance of functional groups which subsequently allow the covalent binding of the antibodies to the surface. The monolayers are formed using hydrocarbon molecules containing functional groups at one or both ends and, additionally, can be auto assembled [15]. The monolayers formed from mercaptoundecanoic acid [16] are representative examples of these structures.

On the other hand, the covalent coupling of the antibody to the surface would enhance its immobilization thus improving its concentration and orientation. For this purpose, −NH_2_, −COOH and -SH groups of the antibodies have been used. However, −NH_2_ and -COOH groups are ubiquitous throughout the antibody structure and their use should hinder the correct antibody orientation [17]. Antibody immobilization through -SH_2_ groups could result in loss of functionality [18] since these groups are exclusively located in the hinge region playing an essential role on the protein tertiary structure.

Other methods have used the affinity of Protein A and G for the Fc (fragment crystallizable region) region of the antibodies, and several surfaces have been developed that facilitate the adequate orientation of the antibodies and thereby improving the sensitivity of the ELISA [19].

Due to its multiple biomedical applications, chitin (poly N-acetyl glucosamine), one of the most abundant polysaccharides in nature, is broadly used in the pharmaceutical industry. The chitin binding domain (ChBD) of *Bacillus circulans* WL-12 chitinase, specifically binds to the insoluble or regenerated form of chitin but not to other polysaccharides such as cellulose, chitosan or starch. This fact results in higher stability and affinity of this union in a wide pH range [20].

To improve the orientation and density of antibodies, and thus the sensitivity of the ELISA, we have used chitin-coated polystyrene surfaces. Chitin was regenerated by acetylation from different chitosan sources “in situ”. These surfaces prepared with acetylated chitosan were used in the determination of human IL-8 as a model of quantification by ELISA. The results show an improved detection limit with the consequent improvement of the ELISA sensitivity, possibly due to an increase immobilization and better orientation of antibodies fused to the chitin binding domain.

## Results

**Fig. 1:**
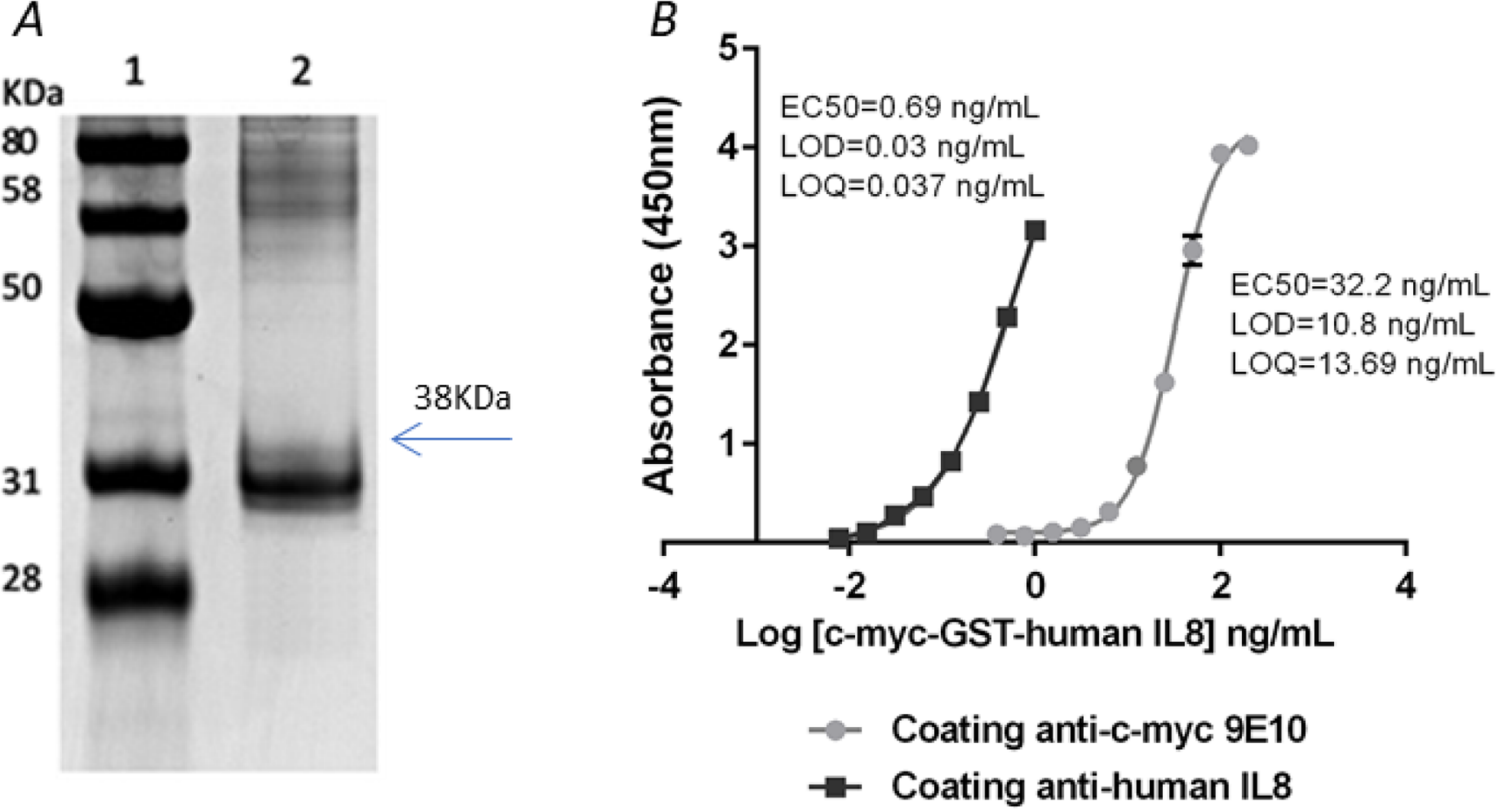
Analysis of His6-c-myc-GST-IL8h recombinant protein obtained in *E. coli*. In panel **a** we show 10% acrylamide gel electrophoresis and Coomassie blue staining. Lanes: 1. Molecular weight pattern (Gene On); 2. His6-c-myc-GST-human IL8 (1 μg). The arrow indicates the analyzed protein with the expected MW. In panel **b** we show the 4PL sigmoidal curve of the ELISA developed using anti-human IL8 or anti-c-myc 9E10 as capture antibody and both revealed using anti- human IL8 biotin. The error bars correspond to three replicates of each concentration of His6-c-myc GST IL8h protein. EC50 represents the analyte concentration capable of generating half of the maximum signal obtained in the test in the linear range of the curve. LOD is the minimum amount of analyte detected and LOQ represents the minimum amount of analyte that can be quantified by the test

Firstly, we obtained a plasmid for the expression of c-myc-GST-IL8h fusion protein. After checking by sequencing, it was used to transform *E. coli* BL21 (DE3) strain. The *E. coli* BL21 (DE3) cultures yielded 10 mg/L of His6-c-myc-GST-IL8h recombinant protein. The electrophoresis gel showed a band of approximately 38 kDa, which correlated well with the expected molecular mass. Also, a high degree of purity of this protein was noticeable (Fig. 1a).

The recombinant protein His6-c-myc-GST-IL8h was detected in the sandwich ELISA using anti-human IL-8, as well as with an anti-c-myc-ChBD as capture antibody (see Fig. 1b). Both ELISAs were developed in parallel on standard unmodified surfaces. Figure 1b shows the differences observed in the dynamic range of concentrations. When using standard surfaces coated with the human anti-IL-8 antibody, ELISA LOD and LOQ were 0.03 and 0.037 ng/mL, respectively. However, when coating with the anti-c-myc antibody 9E10, these values were 10.8 and 13.69 ng/mL, respectively. Thus, when changing only the capture antibody, the difference between both ELISAs was between 360 and 370 times more sensitive.

**Fig. 2:**
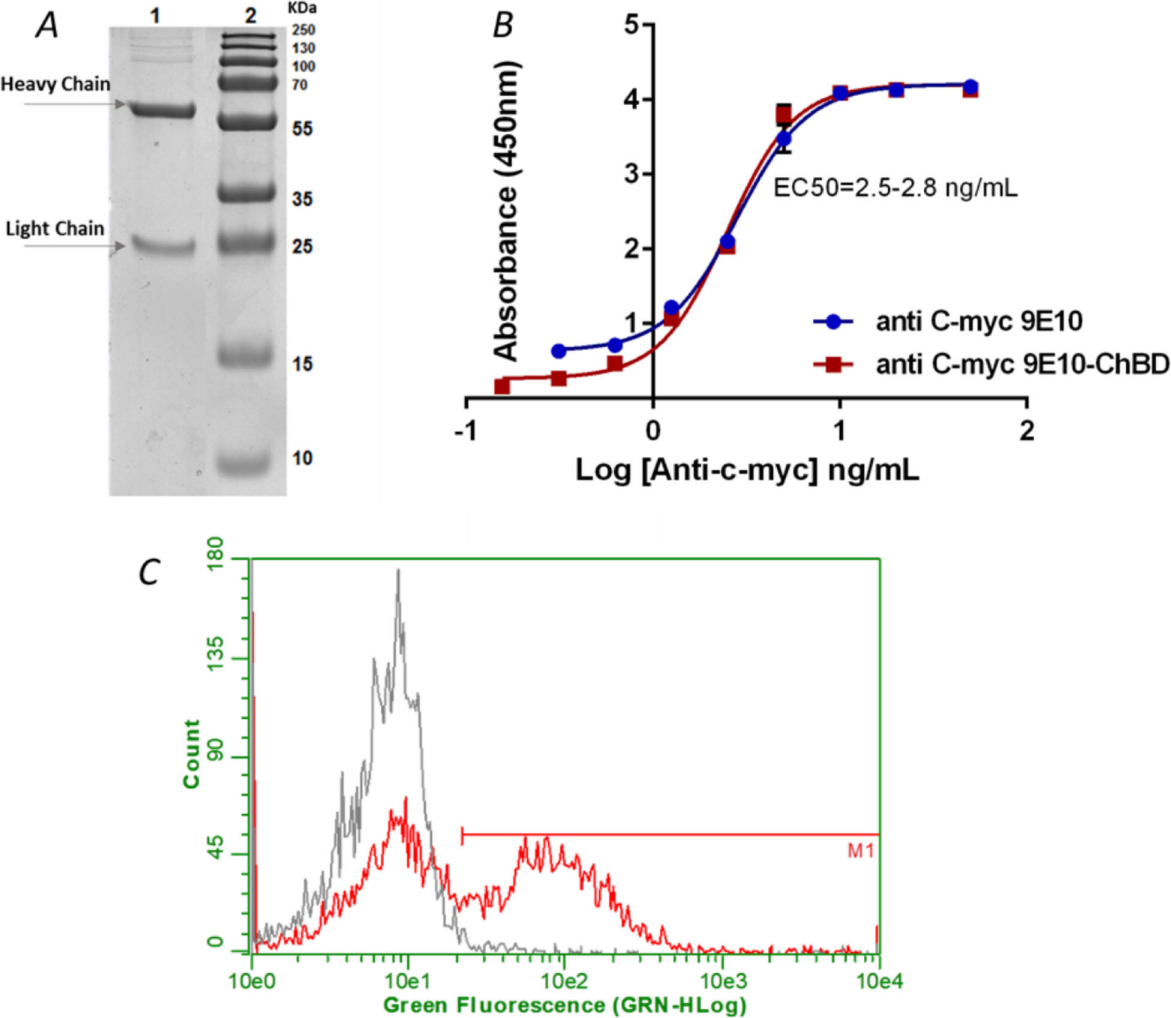
Analysis of the anti c-myc-ChBD antibody expressed in mammalian cells. Panel **a** shows 10% acrylamide gel electrophoresis and Coomassie blue staining. Lanes: 1. anti c-myc- ChBD with β Mercaptoethanol (2 μg); 2. Molecular weight pattern. Panel **b** shows the ELISA detection of anti-c-myc-ChBD antibody activity compared to the antibody purified from hybridoma 9E10. In panel **c** cytometry of chitin-coated magnetic particles using the specific anti-c-myc-ChBD antibody is depicted. The negative cytometry control for which we used the anti-c-myc antibody is represented in black; particles tagged with the recombinant anti-c-myc-ChBD antibody are represented in red

Using a standard protocol, the anti-c-myc-ChBD antibody was expressed with the ExpiCHO system. This antibody had an expression of 40 μg/mL, and the affinity purification with Protein G column using the AKTA system, allowed the isolation of the pure antibody (Fig. 2a). It specifically recognized its antigen with the same sensitivity than the unmodified anti-c-myc 9E10 antibody, as confirmed by a direct ELISA (Fig. 2b). Flow cytometry on chitin particles showed that the chitin binding domain of chitinase A1 from *Bacillus circulans* WL-12 fused to the anti-c-myc antibody, was active and capable of binding to chitin in a specific manner. Its union to 50% of the particles was detected (Fig. 2c).

**Fig. 3:**
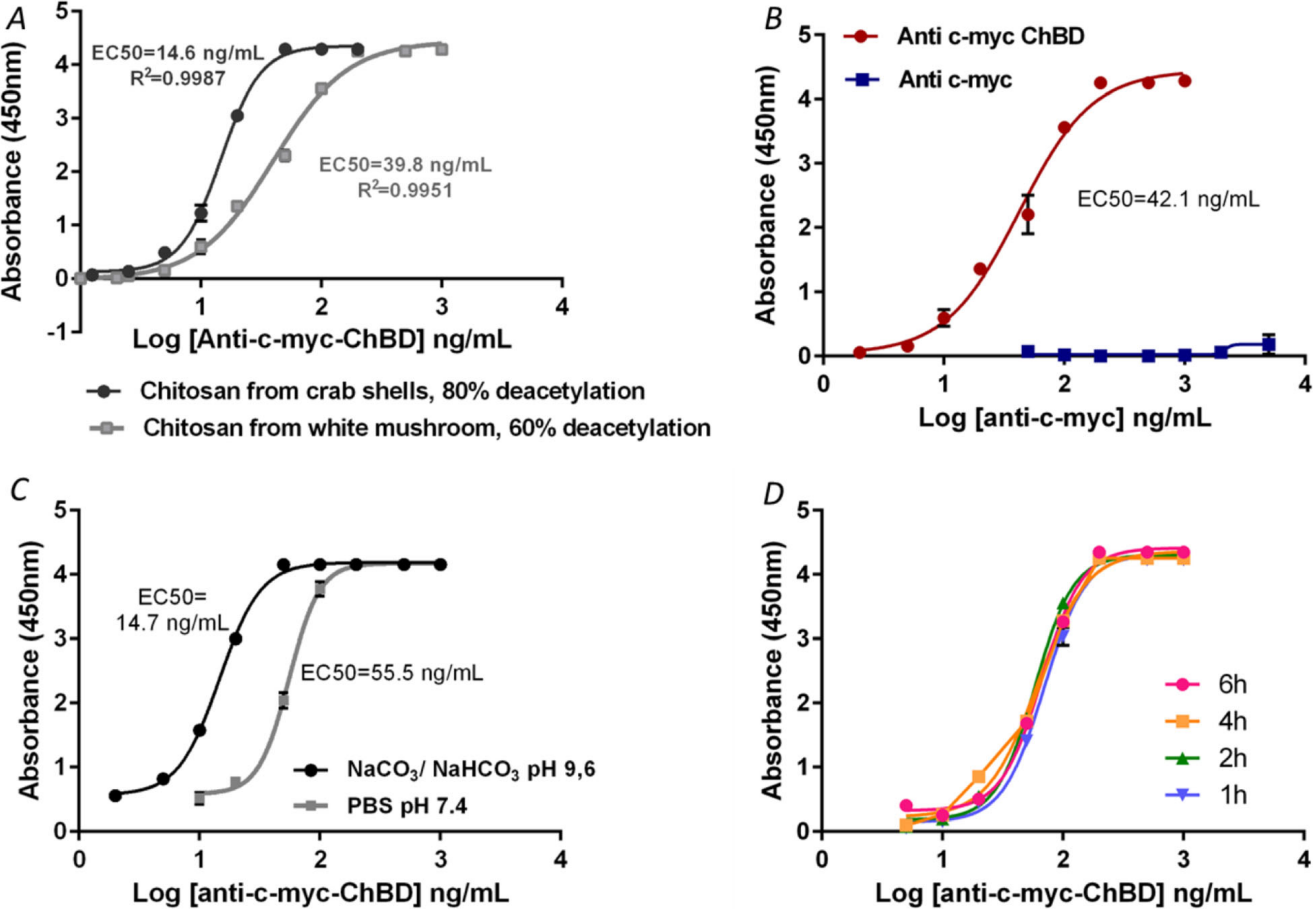
ELISA conditions establishment in acetylated chitosan surfaces. Panel **a**: Comparison of acetylated chitosan surfaces: anti-c-myc-ChBD antibody binding ELISA on surfaces prepared with chitosan at different degrees of deacetylation. Panel **b**: anti-c-myc-ChBD binding curve to chitin modified surfaces as a negative control anti c-myc 9E10. Panel **c**: Binding curves of anti-c-myc-ChBD to chitin surface in different buffers. Panel **d**: anti-c-myc-ChBD binding curve to chitin at 37 °C and using different incubation times. All charts show error bars of 3 replicas for each concentration

Chitin plates with two types of commercial chitosan were prepared, one with 60% deacetylation and the other with 80%. Both plates were treated by an “in situ” acetylation process. It should be expected that lower percentage of deacetylation would produce better results. However, the signal was higher in those plates prepared from chitosan with 80% deacetylation (Fig. 3a). Besides, when the absorbance and the coefficient of variation of 10 negative control wells were analyzed, these parameters were much higher in acetylated chitosan prepared from a chitosan with 60% deacetylation (Mean blank = 0.208; %CV Blank = 18.66) than in the surfaces prepared from chitosan with 80% deacetylation (Mean blank = 0.170; %CV Blank = 4.70). This result is probably due to the fact that 80% deacetylated chitosan surfaces are less viscous than those prepared using solutions of chitosan with 60% of deacetylation. Therefore, the 80% deacetylated chitosan was selected for the preparation of chitin plates.

As we show here, the binding to surfaces coated with insoluble chitins specific and is recognized only by an antibody fused to the ChBD. Accordingly, the anti-c-myc-ChBD antibody bound specifically to the chitin surface, and no binding of the same antibody lacking the ChBD domain was detected (Fig. 3b). This ELISA was repeated 5 times on different days, and it was determined that in the 95% confidence interval, the EC50 ranged from 47.54 to 67.22 ng/mL. As this is the capture antibody, its concentration should be saturating in the sandwich ELISA, and then the EC95 is calculated to use a higher concentration. The EC95 range was 155.41 to 219.75 ng/mL.

To optimize the binding reaction conditions of ChBD protein with the chitin surface, we performed several tests to improve the experimental conditions (reaction buffer and incubation time of the ChBD-chitin binding). The test buffer selected was NaHCO_3_ since the EC50 was 14.7 ng/mL, lower than that obtained with PBS, which was 55.5 ng/mL (Fig. 3c). After one-hour incubation, no signal variation was detected. Thus, one hour was selected as the incubation time for binding of anti-c-myc-ChBD antibody to the chitin surface (Fig. 3d).

**Fig. 4:**
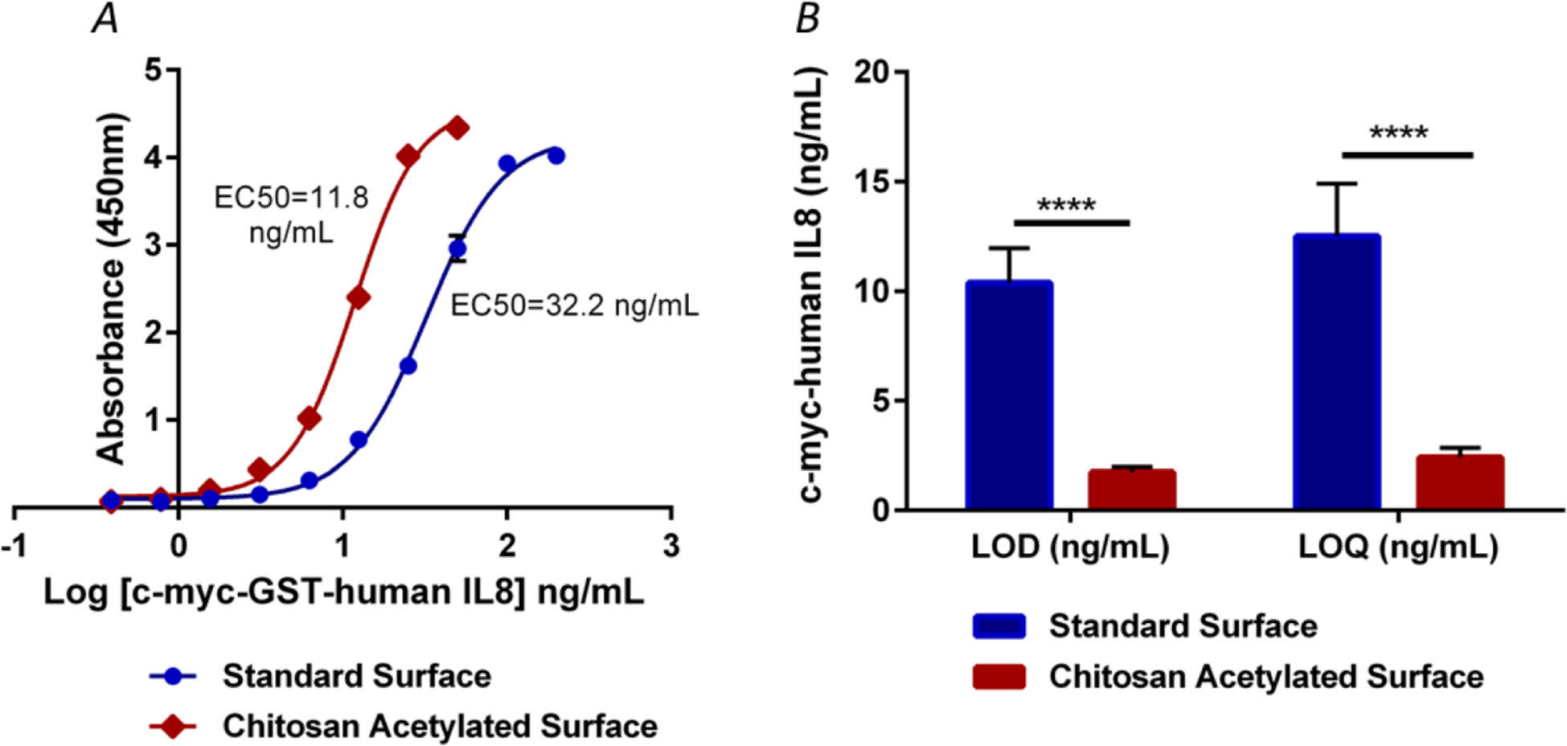
Comparative ELISA of human c-myc-GST-IL8 in standard and chitin-treated plates. Panel **a**: 4PL sigmoidal curves on two different surfaces developed ELISAs. Concentration range used was 0.39–200 ng/mL. The results correspond to three replicates for each concentration. The negative control included 10 replicas. Panel **b**: bar diagram of LOD and the resulting LOQ of five ELISAs on each surface. The value of p was determined using the GraphPad Prism software

ELISAs performed on chitosan plates using anti-c-myc-ChBD as capture antibody and ELISAs developed on standard plates using an anti-c-myc antibody were compared (see Fig. 4). The results show that the ELISA performed on chitin surface has a shift of the sigmoid curve to the left, indicating that lower concentrations of the analyte, were detected and quantified. In fact, on the modified surface we detected analyte concentrations that cannot be measured using standard plates (Fig. 4a).

In the standard plates, the minimum detected amount of human His6-c-myc-GST-IL8h protein was 10.4 ng/mL, while in the chitin surfaces it was 1.74 ng/ml, with 6-fold increase of the detection limit (Table 1). Also, the lowest quantified amounts were 12.5 and 2.4 ng/mL respectively, a 5.2 fold improvement of the detection limit. The statistical analysis yielded significant differences in both the LOD and the LOQ between ELISAs (*p* < 0.0001 in both cases; see also Fig. 4b).

**Table 1:** Comparison of the obtained results for standard and acetylated chitosan surfaces

	High binding surfaces	Chitosan acetylated surfaces
LOD (ng/ml)	10.38 ± 1.58	1.74 ± 0.23
LOQ (ng/ml)	12.50 ± 1.41	2.40 ± 0.34
%CV Intra-assay	0.27–6.05	0.17–4.90
%CV Inter-assay	0.77–8.35	1.37–6.59

LOD: Limit of detection; LOQ: Limit of quantification; CV: Coefficient of variation.

During ELISA assay development a Spike/Recovery assessment is commonly performed to determine whether the value obtained from a sample is accurate and reliable or if there is some factor in the natural sample matrix interfering with the measurement. In this assay, a known amount of recombinant protein, in this case His6-c-myc-GST-IL8h, is “spiked” into a sample and measured in the ELISA. The resulting concentration should not differ significantly from the expected value of the spiked concentration. If there are differences, some factor in the sample may be inhibiting the detection of the protein in this system.

**Fig. 5:**
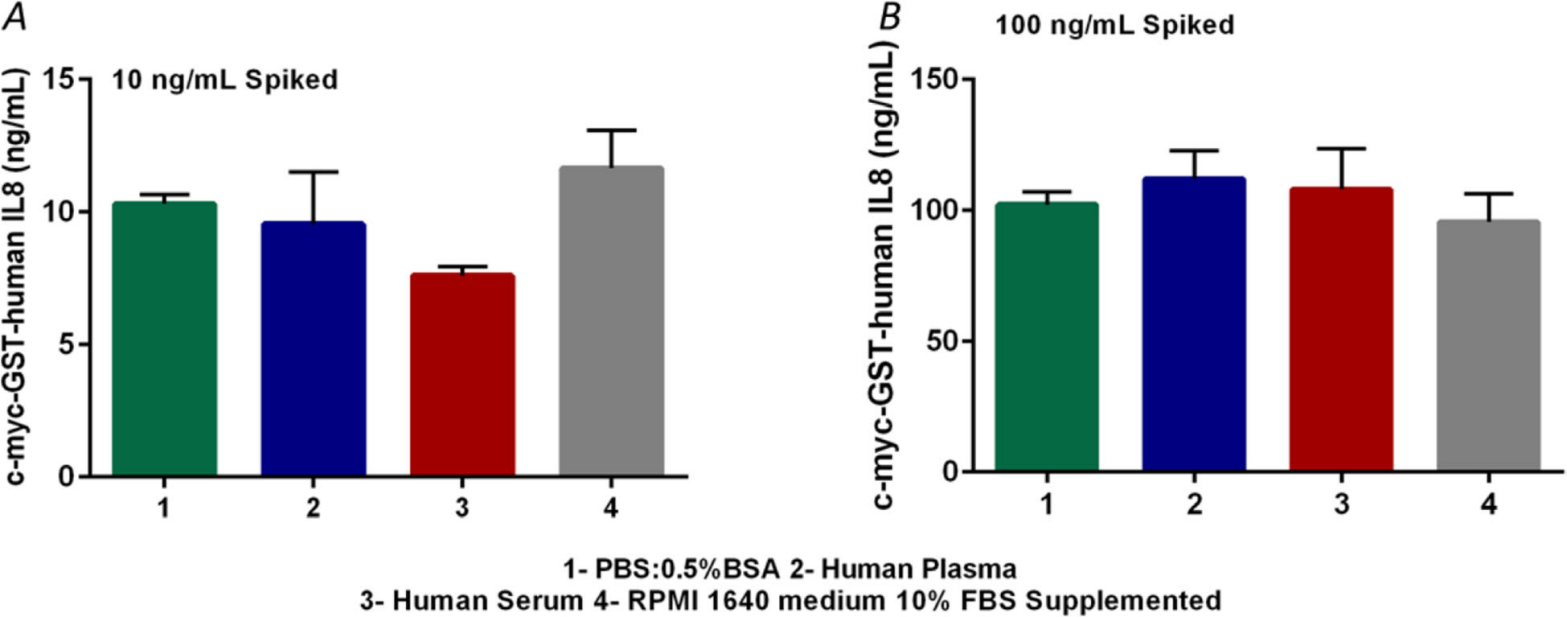
Detection of His6-c-myc-GST-IL8h protein in plates prepared with chitin and using different concentrations of a biological sample. Panel **a**: Bar graph of the resulting ELISA concentration when the protein to be analyzed was added at final concentrations of 10 ng/mL **b**: Bar graph of the resulting ELISA concentration when the protein to be analyzed was added at final concentrations of 100 ng/mL

His6-c-myc-GST-IL8h protein was added to human serum and plasma samples, to RPMI medium plus 10% FBS and to PBS: BSA 0.5% at 10 and 100 ng/mL final concentration. The protein was then measured on each sample and the concentrations were calculated using a standard curve. The protein concentrations in PBS: BSA 0.5% was taken as 100% of recovery because this buffer is commonly used to elaborate standard curves (see Fig. 5 a-b). The recovery acceptance ranges were 80–120% (*R&D Systems*). As observed, excepting the 73% recovery, the other percentages fit with the expected values (Table 2). These results indicate that chitin-prepared plates can be used for the detection of proteins in samples from patients as well as from cell cultures.

**Table 2:** ELISA analyte recovery in different media compared to the control

Sample	Spiked level (ng/ml)	ELISA results (ng/ml)	%Recovery
Human Plasma	10	9.54	92.54
	100	112.11	109.73
Human Serum	10	7.59	73.69
	100	108.00	105.70
RPMI medium (10% FBS Supplemented)	10	11.66	113.15
	100	95.53	93.50

## Discusion

ELISA developed with different capture antibody, in this work, anti human-IL8 or anti c-myc 9E10 antibody to detect recombinant protein His6-c-myc-GST-IL8h, show differences in the dynamic range of concentrations. These results were due probably to the sensitivity of the assay, since coating with the anti-c-myc antibody is less sensitive than coating with the anti-human IL8 antibody. This reveals that coating with low affinity antibodies, ELISA displays a lower sensitivity for analyte detection.

When an antibody is used for detection or quantification of its antigen, the assay sensitivity greatly depends on the dissociation constant (Kd) of the antibody with its antigen: if the antigen-antibody interaction is weak, Kd value is high, while if the affinity is high, Kd value is low. In agreement with most of the reports, the antibody-antigen binding constant may vary in a wide range from 10^− 5^ to 10^− 12^ M^− 1^ [21]. The anti-cytokines binding constant is about picomolar range while anti c-myc antibody monoclonal (clone 9E10) has low affinity in at least two order of magnitude to its antigen, Kd = 5.3 ± 0.3 × 10^− 7^ M [22, 23]. If the binding constant increases by two orders of magnitude, the detection limit can reach 1 pg/mL [24].

Chitin is an insoluble linear β-1,4-linked homopolymer of N-acetyl-glucosamine. Its solubilization with alkali results in chitosan, which forms films whose amino groups, can be readily acetylated with acetic anhydride treatment [25]. This acetylation is necessary since ChBD only recognizes insoluble chitin but no other polysaccharides such as chitosan, cellulose or starch [20]. Once chitosan is acetylated, ChBD specifically binds to it.

Here we show that the binding of the antibody fused to ChBD to acetylated chitin surfaces is specific. Using neutral or alkaline pH reaction buffer, we obtained better results at higher pH. This result agrees with those reported by Hashimoto et al. [20] since they demonstrated that ChBD from chitinase A1 shows binding activity over a broad range of pHs with the highest binding at pH 9.

Bernard et al. [26] used surfaces coated with acetylated chitosan to detect a ChBD-tagged heterodimeric human glycoprotein hormone analog directly from mammalian cell culture media. They reported that the binding to the surface was stable in sodium dodecyl sulfate and only partially reversed at low pH or in 8 M urea at 37 °C. These facts indicate that ChBD- chitin binding is useful and suitable for ELISA development in those prepared surfaces because of the high ChBD and chitin binding stability. However, these authors did not develop an ELISA to bind an antibody to acetylated chitosan surface.

In our assay we used acetylated surfaces not only to detect ChBD-tagged protein but also to improve the detection of any molecule by immunoassay. Antibodies fused to ChBD enabled us to perform any type of ELISA: sandwich, competitive or indirect. In contrast to this, the method described by Bernard et al. [26] is restricted to analytes tagged with the ChBD. In other hand, they used radioactive readout by using radiolabeled monoclonal antibodies. We reported in our work the coupling of acetylated chitosan surface to a colorimetric enzymatic assay.

In biomolecular assays and sensor development, the detection limit is of capital importance and, consequently, numerous investigations to bring the detection limit of bioanalytical techniques to the lowest possible levels have been carried out. In ELISA assays the orientation of immobilized antibodies to improve immunoassays has been extensively investigated [14].

To promote site directed antibody binding, affinity immobilization techniques are good strategies [27]. Among others, these techniques use the affinity of antibodies for Protein A (a surface protein of *Streptococcus*) and Protein G (a surface protein of *Staphylococcus aureus*) [18, 28], specific peptides and aptamers that bind to antibodies Fc region [11, 29] and the biotin-streptavidin interaction [30].

The interaction between streptavidin-coated surfaces and biotinylated antibodies do not support the correct orientation of the antibodies because biotinylation is a random process. The biotin can attach to the amino group of any lysine and also to an amino terminal. Consequently, the antibody may not be properly oriented on the surface. But by far the most important limitation of this procedure is that generally biotinylated antibodies are used as secondary antibodies, that is, antibodies that are used to detect the analyte bound to a first coating antibody. Cho et al. [31] conjugated antibodies to biotin at the hinge disulfides and found only two-fold improvement in the antigen detection relative to the system using random biotinylation of antibodies. Antibodies immobilization using coating microplates with G protein is a commercial technique and there have been attempts to improve its outcomes [19]. Nevertheless, it has the disadvantage that the use of IgG as secondary antibodies must be avoided as any free G protein will react with such secondary IgG. This fact is very important as most commercial antibodies used today in ELISA are IgG isotype.

As we demonstrated, a recombinant antibody fused to ChBD by the Fc region would be an excellent method to achieve adequate orientation of antibodies in ELISA microplates similar to trapping antibodies with Protein A or G. This system based on acetylated chitosan surface-ChBD antibody has the additional advantage that all antibody isotypes and any biotinylated antibody can be used for detection.

The employment of chitosan acetylated surfaces and antibodies fused to CHBD greatly increase the ELISA sensitivity through the enhancement of the detection limit. Probably, the improvement is achieved by the adequate orientation of antibodies in the microplates well used in our system. The literature cited in this manuscript shows that the binding to polystyrene surfaces tends to distort some trapping antibodies enough to destroy their value as a capture antibodies [12, 13]. The method described in this paper avoids that tendency because the binding to surface is through affinity and not by hydrophobic and electrostatic interactions.

## Conclusions

In summary, the data showed in this work support that acetylated chitosan surfaces improve biomarkers detection even using low affinity antibodies. ELISA developed on chitin surface was 6-fold more sensitive than those performed on standard surfaces. This method has the advantage that can be developed for any biomarker and any antibody isotype can be used as a detection reagent. The method should be used to quantify a biological sample that has a concentration below the detection limit of traditional ELISA. Given the increase in sensibility, stability in time and the low cost of its preparation, acetylated chitosan surfaces may be an excellent alternative to standard surfaces.

## Methods

### Construction of the pET43a-His6-c-myc-GST- human IL-8 expression plasmid

The synthetic fragment His6-c-myc-GST-human-IL-8 was cloned into the pET43a vector (Novagen) with the restriction enzymes *Nde*I and *Sal*I. The chimeric protein was constituted by a six histidine tag, one additional tag derived from a terminal fragment of the proto oncogene c-myc (EQKLISEEDL), the glutation-S-transferase protein (GST) and the human cytokine interleukin-8 (Gb: NM_00584.4). The sequence of the recombinant plasmid was confirmed by restriction digestion and DNA sequencing.

### Induction, expression and purification of protein His6-c-myc-GST- human IL-8

*E. coli* BL21 (DE3) cells carrying the recombinant plasmid pET43a-His6-c-myc-GST-human IL-8 were grown in 0.5 L Luria Bertani (LB) medium containing 100 μg/mL of ampicillin at 37 °C. When the bacterial growth reached optical density of 0.8 at 600 nm (OD_600 nm_), 1 mM isopropyl-β-D-thiogalactopyranoside (IPTG) was added to the culture to induce expression of the protein. The culture was incubated for a further 4 h at 37 °C. Then cells were collected by centrifugation (5000 x g for 5 min), washed with PBS supplemented with 1 mM EDTA and resuspended in the same buffer. The cells were disrupted by sonication with a Bandelin Sonoplus disruptor set at 70% of power. The soluble fraction was clarified by centrifugation (4 °C, 14000 x g, 30 min) and was filtered through 0.22 μm filter. Then it was loaded on a 1 mL HisTrap HP column (GE Healthcare 17–5247-01) connected to an AKTA Prime plus (GE Healthcare). After washing with 20 mM sodium phosphate pH 7.4, proteins were eluted by applying the same buffer plus 500 mM imidazole. Protein concentration was determined by the Bradford assay (Bio-Rad) using bovine serum albumin (BSA) as standard. Sodium dodecyl sulfate (SDS) -polyacrylamide gel electrophoresis with 12% polyacrylamide was carried out.

### Comparative ELISA assays for the detection of bi-specific model c-myc-GST-human IL-8 protein using two coating antibodies with different affinity

Maxisorp plates (Thermo 442,404) were coated with the antibodies anti-human IL-8 clone MT8H6 or anti-c-myc purified from 9E10 hybridoma diluted to 2 μg/mL each other in Na_2_CO_3_/NaHCO_3_ pH 9,6 and incubated overnight at 4 °C. After blocking with PBS pH 7 containing 1% (w/v) BSA, the model protein GST-c-myc-human IL-8 was added in serially dilutions from 4 to 1000 pg/mL in the anti-human IL-8 plates and from 0.4 to 200 ng/mL on anti-cmyc coated plates. The next steps were as those described for ELISA as follow. Both ELISAs were developed using an anti-human IL-8 biotin clone MT8F19 antibody at 1 μg/mL. After 5 washing steps 100 μl per well of streptavidin-HRP diluted at 100 ng/mL and TMB (3,3′,5,5′- tetramethylbenzidine) were sequentially added to them. Enzyme reaction was stopped with 1 M HCl and color development was measured at 450 nm on a microplate reader (FLUOstar OPTIMA, BMG Labtech).

### Chimeric antibody anti c-myc-ChBD

#### Cloning of anti-cmyc 9E10 antibody from hybridoma cells and fusion with ChBD

RNA was isolated from 10^6^ cells 9E10 hybridoma using the PRImeZOL™ Reagent (Canvax AN1100) following the manufacture’s protocol, reverse-transcribed (RT) into cDNA and VH and VL genes was amplified by PCR with the high-fidelity DNA polymerase (Canvax P0032). Retro transcription of the fragment VK-CK was performed with the primer pCK-2rev (5’TATGCGGCCGCCTTTGTCTCTAACACTCATTCCTG 3′). The primers pVK-1fw (5’GGGGATATCCACCATGGAGACAGACACACTCCTGCTAT 3′) and pCK-2rev were used for the amplification. On the other hand, VH fragment cDNA was obtained using the antisense primer pVH9E10-6rev (5’TTTTAGATCTAATTTTCTTGTCCACCTTGGTGC 3′) and the elongation was developed with pVH9E10-4fw (5’TTTAAGCTTCGCCACCATGAACTTCGGGCTCAG 3′) and pVH9E10-6rev. PCR products were cloned into pSPARK® vector (Canvax C0001). The pSPARK® vector used for this purpose encoded the LacZ α-peptide when no insert was present. This allowed a blue/white selection on IPTG and X-gal containing LB-agar plates. The funtional obtained sequences were then subcloned into mammalian expression vector pCDNA3.4 driven by cytomegalovirus promoter. The VH fragment cloned was fused to the murine FC IgG.

ChBD (chitin binding domain) of chitinase A1 from *Bacillus circulans* WL-12 was synthetized with the flank restriction enzymes 5′*Nhe*I/ 3′*Not*I. The fragment was ligated simultaneously with the PCR fragment performed with the primers pVH9E10-4fw and pIgG1m-25rev (5’GATAGCTAGCTTTACCAGGAGAGTGGGAGAG 3′) on the pcDNA3.4 vector coding the VH fragment digested by the restriction enzymes *Hind*III/ *Nhe*I. Both fragments were ligated at once into the expression vector opened with the restriction enzymes *Hind*III/ *Not*I.

#### Expression and purification of anti c-myc- ChBD antibody in mammalian cells

Both VH-IgG1 and VL-CK chain of the anti c-myc-ChBD expression vectors were co-transfected in CHO-S (Chinese hamster ovary) cells using the ExpiCHO Expression system kit (Thermo A29133). The antibody was expressed at 32 °C and 5% CO_2_ for 10 days attending to the manufacturer’s instructions. Cells were isolated by centrifugation and the supernatant was measured by mouse IgG sandwich ELISA and purified by affinity chromatography with protein G column (HiTrap Protein G, GE Healthcare 29–0485-81). Pure antibody concentration was measured by absorbance at 280 nm and purity grade was analyzed by SDS-PAGE.

#### Flow cytometry to detect chitin binding domain activity using chitin magnetic beads

For the activity detection of the chitin binding domain fused to the heavy chain of anti c-myc antibody, chitin magnetic beads were used (BioLabs E8036S), following the manufacture’s guidelines. The antibodies were diluted in binding buffer at 1 μg/mL (500 mM NaCl, 20 mM Tris HCl, 1 mM EDTA, 0.05% Triton-X100, pH 8) and incubated with 50 μl of beads at 4 °C for 1 h. After 3 washing steps with the same buffer, 1 μg/mL of anti-mouse FITC was added and incubated at 4 °C for 30 min. Beads were analyzed on a flow cytometer (Guava EasyCyte mini, Guava Technologies).

#### ELISA to detect anti c-myc-ChBD antibody activity compared with the antibody 9E10 from hybridoma

A microplate coated with 2 μg/mL c-myc-GST-human IL-8 antigen was blocked with PBS containing 1% (w/v) BSA. Then serially dilution of the anti-c-myc-ChBD antibody from CHO-S or anti c-myc 9E10 antibody from hybridoma were added and incubated at 37 °C for 1 h. Both antibodies concentrations were ranging from 100 to 0.078 ng/mL. After microplates washing, anti-mouse HRP (Sigma A2554) and TMB were sequentially added to them. Enzyme reaction was stopped with 1 M HCl and color development was measured at 450 nm on a microtiter reader.

### Preparation of microtiter plates with acetylated chitosan

The procedure described by Bernard et al., 2004 with modifications was used. Deacetylated chitosan (30 mg) were dissolved in 5 mL of 0.1 M Sodium acetate pH 3. Two deacetylated chitosans with different grades of deacetylation were used: chitosan from white mushroom (60% deacetylation, Sigma 740,063) and chitosan from crab shells (80% deacetylation, Sigma 48,165). Each solution was diluted 20-fold in sodium acetate pH 5, and the polystyrene plates filled with 50 μL per well. Afterward, 8 μL per well of acetic anhydride (Sigma 320,102) were added. The plate was placed in a fume hood and allowed to dry overnight. Eventually, the plate was washed using PBS 10X pH 7.4 and blocked with 0.3 mL PBS (10x) pH 7.4: BSA (1 μg/mL).

#### Comparison of microplates coated with two different deacetylated chitosans by binding of the chimeric anti-c-myc-ChBD antibody

Two different chitosan acetylated microplates prepared using white mushroom and crab shells chitosan. Anti-cmyc-ChBD antibody in serial dilutions ranging from 0.039 to 50 ng/mL were added. Microplates were incubated at 37 °C for 2 h. After washing, anti-mouse HRP (Sigma A2554) and TMB were sequentially added to them. Enzyme reaction was stopped with 1 M HCl and color development was measured at 450 nm on a microtiter reader.

### Optimization of protein c-myc-GST-human IL-8 detection in acetylated chitosan microplates

Anti c-myc-ChBD antibody concentration, incubation time (1, 2, 4, 8 and 16 h) and the binding buffer for the binding reaction chitin- chitin binding domain were established. Concentrations ranging 0.312 to 1 μg/mL of anti c-myc-ChBD antibody and PBS pH 7.4 or NaHCO_3_ pH 9 buffers were used. ELISA assays were carried out on chitosan acetylated surfaces. Anti c-myc antibody from 9E10 hybridoma was used as negative control of the chitin specific binding. ELISA was revealed with an anti-mouse HRP (Sigma A2554).

### Comparative ELISA between standard polystyrene and chitosan acetylated surfaces

**Fig. 6:**
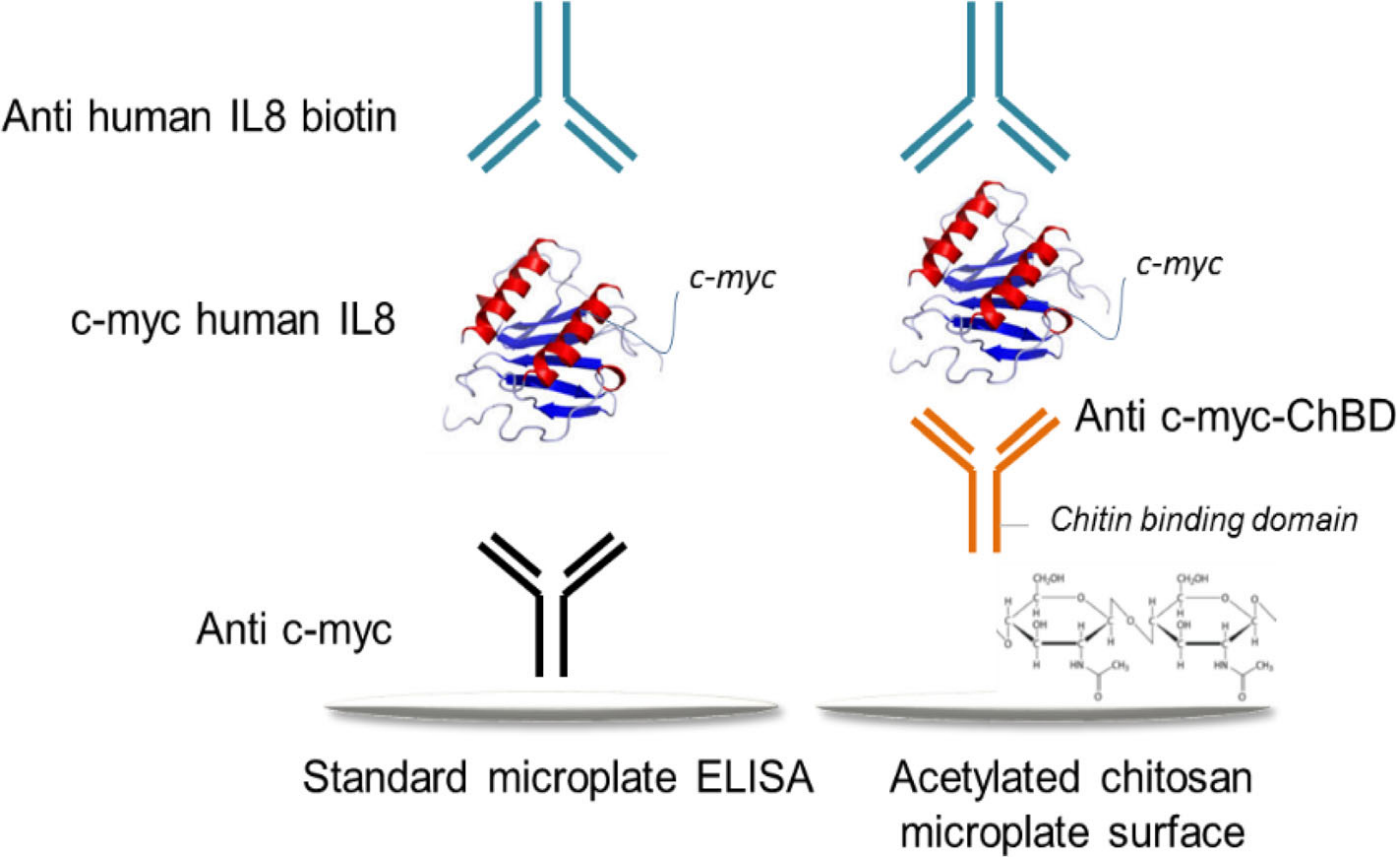
Schematic illustration of a comparative ELISA developed in standard or chitosan acetylated microplates

ELISA assays were performed simultaneously in standard polystyrene (Maxisorp, Thermo 442,404) and in chitosan acetylated microplates (Fig. 6). Standard microplates were coated with the anti-c-myc antibody while chitosan acetylated plates were coated with the anti-c-myc-ChBD antibody. Afterwards, a concentration range of the chimeric protein His6-c-myc-GST- human IL-8 from 100 to 0.39 ng/mL, was prepared. 100 μL of samples per well were added and incubated at 37 °C for 1 h. After washing steps, the biotinylated antibody anti IL-8 (clone MT8F19) and streptavidin- conjugated horseradish peroxidase were used. TMB was added for color development and plates were measured at 450 nm on a microtiter reader. Both ELISAs were performed simultaneously under the same conditions, on 5 different days, using three replicates of each concentration and 10 replicates of the target.

#### Influence of serum, plasma and cell culture medium using acetylated chitosan microplates

The c-myc-GST-human IL-8 spiked samples were prepared by mixing two different concentrations of protein, 100 ng/mL and 10 ng/mL in human serum, human plasma, PBS: BSA 0.5% and cell culture medium (RPMI 1640 medium). The sample concentrations were determined in chitosan acetylated microplates plotting in control curve performed in PBS-BSA 0.5%.

The recovery percentages were calculated using the concentrations measured in PBS: BSA as 100% of recovery or expected concentration. We used the equation:

% recovery = (Sample concentration observed- medium concentration unspiked)/ Concentration obtained in PBS: BSA 0.5%.

### Assay performance analysis

All datasets obtained from the developed ELISA procedures were subjected to four-parameter logistic function- based standard curve analysis in the GraphPad Prism (GraphPad Software Inc., San Diego, CA, USA): EC50, R^2^, to determine limit of detection (LOD) and limit of quantitation (LOQ). The EC50 indicates the concentration at which half of the test signal is obtained or the half maximal effective concentration, and R-squared, is a statistical measure of how close the data are to the fitted curve. Both parameters were determined from the report data generated by the software.

Absorbance corresponding to the detection limit was the result of the average absorbance of the blank from all the repeats plus 3 standard deviations of the blank while for quantification limit was the same but 10 standard deviations. Then, by plotting the absorbance on the standard curve, the values of the LOD and LOQ concentration are obtained.

## Data Availability

The datasets used and analyzed during the current study are available from the corresponding author on reasonable request.

## Abbreviations

ELISA: Enzyme-linked immunosorbent assay
ChBD: Chitin binding domain
Fc: fragment crystallizable
LOD: Limit of detection
LOQ: Limit of quantification
Kd: Dissociation constant
CV: Coefficient of variation
GST: Glutation-S-transferase
IL8: Interleukin-8
BSA: Bovine serum albumin
